# Highly Sensitive Tunable Magnetometer Based on Superconducting Quantum Interference Device

**DOI:** 10.3390/s23073558

**Published:** 2023-03-28

**Authors:** Antonio Vettoliere, Carmine Granata

**Affiliations:** Consiglio Nazionale delle Ricerche, Institute of Applied Sciences and Intelligent Systems, 80078 Pozzuoli, Italy

**Keywords:** SQUID, magnetometer, magnetic field noise, Josephson junctions, integrated device

## Abstract

In the present article, experimental results regarding fully integrated superconducting quantum interference devices (SQUID), including a circuit to tune and optimize the main sensor device characteristics, are reported. We show the possibility of modifying the critical current of a SQUID magnetometer in liquid helium by means of a suitable heating circuit. This allows us to improve the characteristics of the SQUID sensor and in particular to optimize the voltage–magnetic flux characteristic and the relative transfer factor (responsivity) and consequently to also improve the flux and magnetic field noise. It is also possible to reset the SQUID sensor in case of entrapment of magnetic flux, avoiding taking it out of the helium bath. These results are very useful in view of most SQUID applications such as those requiring large multichannel systems in which it is desirable to optimize and eventually reset the magnetic sensors in a simple and effective way.

## 1. Introduction

Nowadays there is a great interest in quantum technologies due to their potential applications ranging from quantum computing, quantum cryptography, to ultra-sensitive sensors [1,2,3]. These applications can be used to study complex systems for future discoveries and further technological advances in many fields such as medicine, biology, chemistry, pharmacology, bioengineering, atmospheric physics, artificial intelligence, transport, etc. As is known, superconductivity is the most extraordinary manifestation of quantum mechanics at the macroscopic level, and superconducting devices are consolidated and tested examples of quantum technologies [1]. In particular, superconducting quantum interference devices (SQUID) are the most sensitive magnetic field and flux sensors, reaching an energy sensitivity per band unit equal to a few Plank constants [4,5,6,7,8]. Such extraordinary sensitivity lies in its quantum nature and in particular in the fact that a macroscopic quantity such as voltage or a current of the order of a few tens of mV or µA is linked to one of the constants of quantum mechanics, i.e., the elementary magnetic flux quantum Φ₀ = h/2e, where h is the Planck’s constant and e is the charge of the electron. It is an extremely small quantity (2.07 × 10^−15^ T·m^2^), so, thanks to suitable low noise readout electronics, it is possible to measure quantities of magnetic flux lower than 1 µΦ₀ or a magnetic field of 1–3 fT per band unit.

Among various magnetic sensors that are used as magnetometers, such as those based on induction coils, parallel or orthogonal fluxgate, the Hall effect, giant magnetoresistance, tunnel magnetoresistance, anisotropic magnetoresistance, and giant magnetoimpedance [9,10], the only ones able to compete in terms of sensitivity with the SQUID magnetometers are the atomic magnetometers [11,12,13]. By using the quantum properties of atoms, these magnetometers are able to reach a magnetic field sensitivity of 7–10 fT per band unit. Even though their sensitivity is smaller than SQUID magnetometers, they do not require to be cooled below the critical temperature of superconductors by liquid helium as in the case of the SQUIDs. However, the current applications that require very high sensitivity still use SQUID based sensors. Among these, it is worth mentioning the biomagnetism, quantum computing, geophysics, magnetic microscopy, nanomagnetism, non-destructive analysis of materials, and fundamental physics experiments [5,6,7,8,14,15,16,17,18,19].

Among the aforementioned applications, biomagnetism is certainly one of the fields where SQUID sensors play a fundamental role providing an effective tool to study the magnetic field related to the electric activity in the human body [20,21]. In particular, magnetoencephalography is a functional investigation of the brain based on the measurement of the very weak magnetic signals generated by neuronal currents whose frequencies range from a few Hz to a few hundred Hz [22,23,24,25]. Additionally, being that these magnetic fields are very small (on the order of 10–100 fT), magnetometers with a magnetic field noise spectral density lower than 5 fT/√Hz and a low-frequency noise knee of a few Hz are required. SQUID sensors, meeting both requirements, are currently used in all magnetoencephalography systems, while the possibility of using atomic magnetometers has been evaluated for some years [26,27].

Practically, a SQUID is a converter of magnetic flux into a voltage having an extremely low magnetic flux noise. By measuring the voltage across the SQUID and knowing the area of the ring, it is possible to determine the magnetic field. Obviously, the larger the ring area of the SQUID, the better its sensitivity in the magnetic field. However, since the magnetic flux noise is directly related to the ring inductance, it is not possible to greatly increase the area of the superconducting ring. So, special configurations are used to increase the magnetic field sensitivity of a SQUID device if it is used as a magnetometer. One of most used configurations exploits a superconducting flux transformer, consisting of a pickup coil (a square or circular single coil) connected in series with a multiturn coil magnetically coupled to the SQUID ring [28,29]. Another possibility is to use a multiloop configuration in which to decrease the total inductance of the SQUID, large enough loops are used in parallel. In this way, the effective area remains quite large and at the same time the total inductance does not degrade the performance of the sensor [30,31].

The performance of a SQUID device depends on some fundamental figures of merit, in many of which the Josephson critical current appears as a parameter. Therefore, a careful design of the device as well as a reliability of the fabrication process are essential in order to maximize the performance of the SQUID device. In spite of that, the SQUID critical current value can be different with respect to the expected one, or more generally a different value is required. So, the possibility to perform fine tuning of the critical current especially when the device is operated in a liquid helium bath could be a powerful tool to optimize the SQUID performance during the measurement.

In this article, the experimental results concerning a tunable highly sensitive SQUID magnetometer which includes an integrated circuit offering the possibility to tune the critical current of the device are presented. Since the critical current decreases as the temperature increases, by using a suitable integrated heater, a localized increase in the temperature has been obtained, lowering the critical current value optimizing the device performance. By using the same heater, the device has been heated up to the critical temperature of the superconductor, carrying out a full reset operation useful in case of magnetic flux entrapped in the SQUID, which degrades the performance of the sensors. By this procedure, the main SQUID features such as the voltage vs. magnetic flux characteristic, voltage responsivity, and spectral density of magnetic field noise have been optimized, showing the possibility to perform a fine tuning of the fundamental parameters.

## 2. Sensor Design, Fabrication Process and Experimental Set-Up

**Figure 1:**
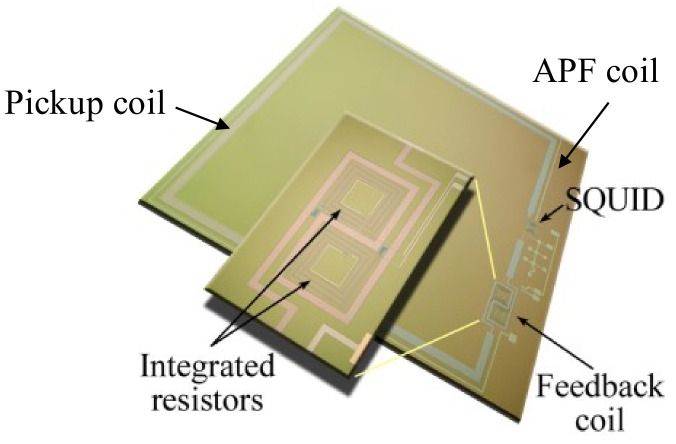
Picture of the fully integrated SQUID magnetometer. The enlarged detail shows the integrated feedback coil consists of two coils wrapped in opposite directions to reduce the crosstalk between neighboring channels and the heating resistors.

The main superconducting elements of the SQUID magnetometer are made of niobium film, while the aluminum oxide is used to realize the Josephson junctions insulating layer, so low critical temperature (LTc) superconducting devices are obtained. It is based on Ketchen-type design, having a superconducting ring in a washer shape in which two Josephson junctions are allocated peripherally and shunted by suitable resistors [29,32]. In such a configuration, the ring inductance does not strongly depend on external dimension, exploiting, at the same time, the flux focusing effect on the washer hole. The magnetometer is provided of a flux transformer in which the magnetic flux is collected by a superconducting pickup coil and transferred to the SQUID ring via a multiturn input coil located under the washer suitably sized (Figure 1). The square pickup coil effective area is *A_p_* = 64 mm^2^ and its inductance is *L_p_* = 27 nH, while the inductance of the input coil, consisting of 12 square turns, is *L_i_* = 33 nH. The SQUID washer one is rather large (*L* = 250 pH) to obtain a high magnetic field sensitivity, that is, the magnetic flux-to-field transfer factor:

(1) $$B_{\Phi }=\frac{1}{A_{\mathrm{eff}}}=\frac{L_{P}+L_{i}}{A_{p}M_{i}}=0.7\frac{nT}{\Phi_{0}}$$

where *M_i_* is the mutual inductance between SQUID and input coil whose value is equal to 2.7 nH. In such a way, an effective flux capture area of 3 mm^2^ is obtained. To prevent a performance degradation due to the increase in inductance parameter (*β_L_*) value, the washer is damped by a suitable resistor. In such a way, on one hand, the effective inductance parameter becomes $\beta_{\mathrm{eff}}=\beta_{L}/\sqrt{1+\beta_{L}^{2}}$, limiting the drawback of relative high SQUID inductance, and on the other hand, the resonance frequencies are shifted out of the working ones.

On the same chip, a circuit for additional positive feedback (APF) [33,34] has been integrated. It consists of a series of a resistor and a coil inductively coupled to the pickup coil that induces an asymmetry on current circulation into the SQUID ring bending the V-Φ characteristics so that the slope locally increases. That is, if the working point is suitably chosen, the voltage responsivity (V_Φ_ = ∂V/∂Φ_e_) increases. Since the SQUID magnetometer is operated in flux locked loop (FLL) mode, the device chip also includes a circuit for negative feedback to null the magnetic flux changes into the SQUID via a current flowing in the feedback coil proportional to SQUID voltage. Such a current is proportional to the applied flux as well as the voltage read across a feedback resistor (27 kΩ).

The feedback coil is made of two multiturn coils wrapped in opposite directions and connected to each other via an integrated resistor (R_h_ = 250 Ω). Such a configuration ensures low crosstalk between neighboring channel in the case of SQUID sensors operating close each to other for example in multichannel systems. The resistor acts as a damping for the resonance phenomena due to feedback inductance and as a heating element to locally increase the temperature to change the SQUID critical current value, if needed.

Furthermore, the heating can be forced to above the critical temperature of the superconductor inducing a temporary transition in the normal state to eliminate the possible magnetic flux entrapped and avoiding the lifting out of the device from the helium bath. This opportunity is particularly useful in multichannel systems in which the sensor heating cannot be performed without warming up the whole system.

The device fabrication process is based on several steps to realize a fully integrated magnetometer including the heating element to improve the SQUID performance. The similar procedure is described in detail in ref. [35]. In the first step, on a silicon wafer provided for the thermal oxide surface layer, the base structure to house the Josephson junctions is patterned by standard optical photolithography on an image reversal resist for high resolution (AZ^®^5214E—MicroChemicals GmbH, Ulm, Germany). On this, a first layer of Nb (base), having a thickness of 200 nm, is deposited by dc sputtering procedure in an ultra-high vacuum system at a pressure of 3 × 10^−8^ torr (4 × 10^−6^ Pascal). Without breaking the vacuum, a successive layer of aluminum, 70 nm thick, is deposited again by dc sputtering by using a second magnetron cathode equipped with an Al target. Then, the surface of this layer is exposed to pure and dry oxygen filling the chamber at a pressure of 188 torr (2.5 × 10^6^ Pascal) to realize a thin insulator barrier made of AlOx by thermal oxidation. After that, the initial vacuum is restored and a further layer of Nb (top), having a thickness of 35 nm, is deposited likewise on the base electrode. After a lift-off procedure, immerging the sample in acetone for 2 h, a patterned Nb/Al-AlOx/Nb multilayer structure is obtained. The Josephson junction realization is made by anodizing of the top Nb layer except for two small areas of 4 × 4 μm^2^ which are covered by a resist layer patterned by optical photolithography to exclude them from the anodization process. In this procedure, performed in an electrolytic solution and where the sample is used as anode while the cathode is a platinum electrode, a current of about 4 mA circulating in the circuit produces the transformation of the metallic Nb in Nb_2_O_5_ oxide. The whole process is monitored by plotting the time rate of oxide growth as a function of voltage across the cell. The process ends when the rate changes abruptly due to the complete anodization of Nb and that of the underlying Al layer begins. To improve the insulation quality at the structure edges and plug possible pinholes, a further insulation layer made of silicon dioxide is added by rf sputtering in a high vacuum system. The lift-off procedure, as described above, concludes this step. The following step consists of the removal of the top Nb layer from the areas dedicated to ensure a clean contact from the final wiring and the base electrode. This is performed by reactive ion etching (RIE) in CF_4_ plasma after the usual, preliminary photolithography step. To realize all the resistors required by the device design, a deposition of a normal metal is made by dc sputtering in ultra-high vacuum conditions. At this step, the Josephson junction shunts, the washer damping, and the set of the resistors of the APF circuit are realized in a gold–palladium alloy having a thickness of 350 nm corresponding to a sheet resistance of 1 Ω/square. At the same stage, the resistor located between the two multiturn feedback coils and used for heating operations is also realized.

In the last step, a thicker Nb layer (500 nm) completes the device, allowing us to realize the SQUID ring (washer), the superconducting APF coil, and all the wiring connections. It is performed in the ultra-high vacuum systems again by dc sputtering deposition at pressure of about 1.0 × 10^−7^ torr (1.3 × 10^−5^ pascal).

The deposition is preceded by a soft cleaning using an Ar+ ion gun to etch the surface oxide formed during the air exposition of the sample. A picture a fully integrated magnetometer is reported in Figure 1. The feedback coil including the integrated heaters is shown in the enlarged detail.

**Figure 2:**
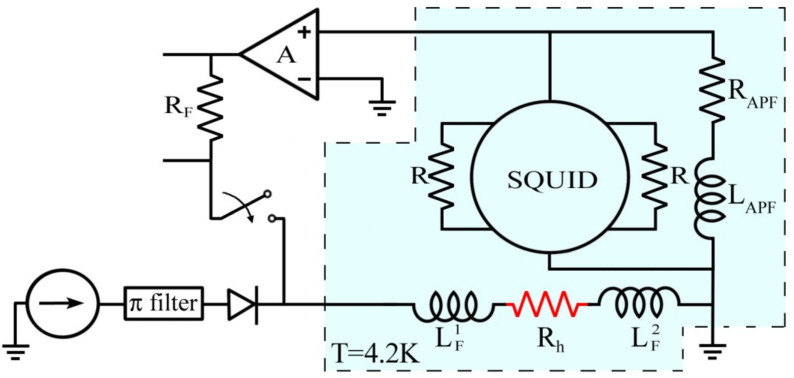
Readout electronic scheme for the SQUID magnetometer characterization. The device is directly coupled to a low noise voltage amplifier. A flux locked loop circuit is used to increase the linear dynamic range. A battery powered current generator sends a current into the integrated heater in order to modify the critical current of the SQUID.

The characterization of the SQUID magnetometer, including the measurements of both voltage as a function of magnetic flux and magnetic flux and field noise in normal and under local heating conditions, has been performed in a helium bath (T = 4.2 K) by using a cryogenic insert equipped with two coaxial cylinders to operate in a high-shielded environment. The inner one is made of superconducting metal (lead), while the outer one consists of a high magnetic permeability material (μ-metal). The measurements have been carried out using a very low noise readout electronics, where the SQUID is direct coupled to a preamplifier and connected to a negative feedback circuit operating in flux locked loop mode to increase the linear dynamic range (Figure 2). The whole amplification stage is integrated on a small standard electronics board located at the top of the cryogenic insert carefully shielded by a copper box and kept at room temperature. All the electrical connections to the SQUID were radio frequency filtered. The current is sent into the integrated heater resistor by a battery powered dc-current generator; a suitable stage of filters is employed to reduce the conducted and/or radiated disturbances through the connecting wires (Figure 2).

## 3. Results and Discussion

**Figure 3:**
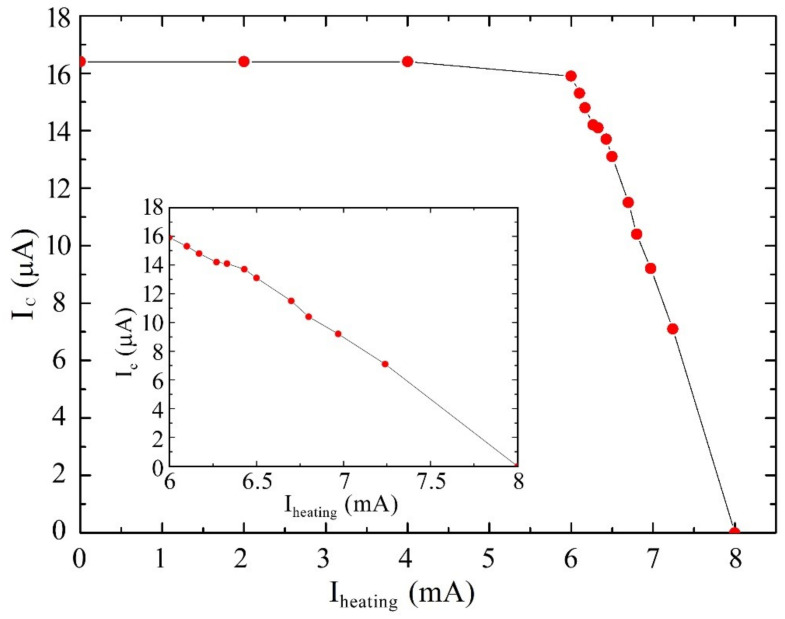
Critical current of the SQUID magnetometer as a function of the current sent into the integrated heater (red dots). After a value of 6 mA, the critical current decreases in appreciable way. The same curve is shown in the inset where the I_heating_ starts from 6 mA to better highlight the behavior of the curve and evaluate the rate of decrease in the critical current. The solid lines are eye-guidelines.

Firstly, the measurement of the dependence of the critical current (I_c_) of SQUID magnetometers on the current value (I_heating_) sent into the integrated heating resistor has been carried out (Figure 3). As it can be seen from the figure, until the heating current is less than 6 mA, the I_c_ of the SQUID does not vary significantly. Once this threshold is exceeded, the I_c_ decreases linearly as the I_heating_ raises with a rate related to the slope of the curve and estimated in about 8 μA/mA. It represents the transfer factor between the heating current and the critical current in the range in which the I_c_ decrease begins to be appreciable. The critical current is completely zero when I_heating_ reaches the value of 8 mA in correspondence of which the temperature of the niobium-based structure of the Josephson junctions reaches its critical value, which is about 8.9 K. So, this procedure can be used also to reset the device in case of magnetic flux trapping by sending a current of 8 mA to the heater for a few seconds ensuring the local transition to the normal state of the superconductor. Of course, these values depend on some conditions such as the resistance value, the substrate properties, and the heater position on the chip. To test the full reversibility, the heating procedure was carried out several times finding the same dependence of the critical current values on the heating ones, and more generally, no changes in the device performance were observed.

The presence of a heating current threshold reflects the fact that the behavior of the critical current of a niobium Josephson junction as a function of the temperature does not vary significantly up to about 6 K, in accord with theoretical prediction, and then it decreases appreciably as the temperature increases.

From the inset of the Figure 3 it is evident that a feedback current variation of about 100 μA may cause an appreciable variation of the critical current leading to a sort of nonlinearity of the flux-feedback response. However, as reported in Equation (1), the magnetic flux-to-field transfer factor B_Φ_ is 0.7 nT/Φ_0_, while the measured feedback current needed to compensate for a Φ_0_ is 10 μA, so the measurement of a magnetic field of 1 nT in FLL mode requires a feedback current of about 14 μA. Therefore, up to about ten nT, the aforementioned effect is not appreciable. Typically, the fields measured by these types of magnetometers in biomagnetic systems are less than one nT, and therefore nonlinearity effects of the feedback response are not observed.

**Figure 4:**
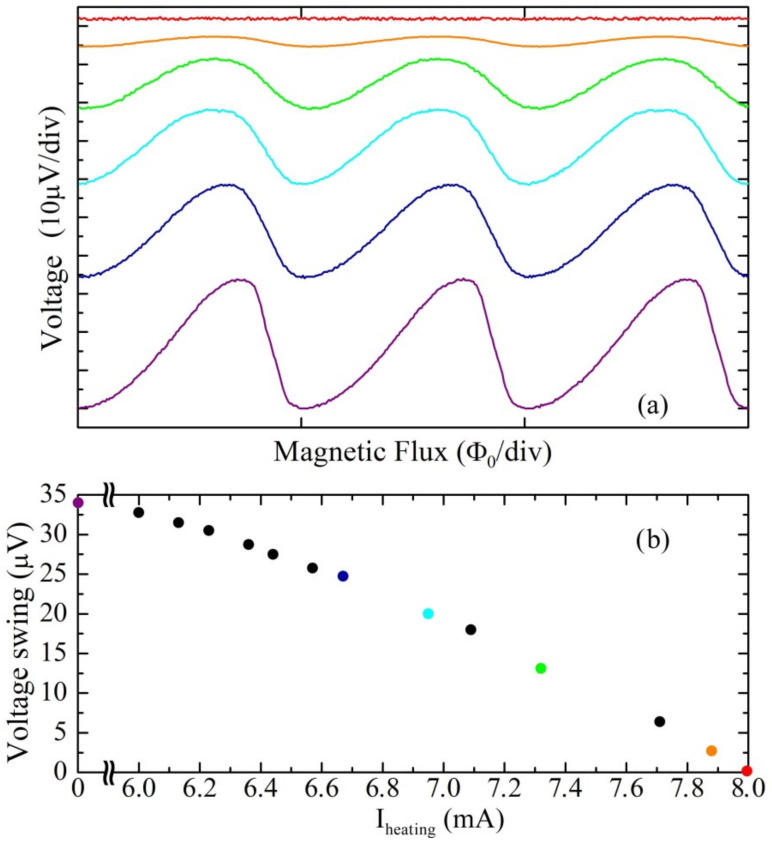
(**a**) Voltage–magnetic flux characteristic (V-Φ) of a SQUID magnetometer for different heating current measured at liquid helium temperature. (**b**) Amplitude of the voltage swing for different heating currents. The color of each V-Φ curve reported in (**a**) corresponds to the circle with the same color in (**b**).

Figure 4a shows the voltage behaviors as a function of external magnetic flux of the same SQUID magnetometer for different values of heating current. The bottom curve corresponds to a zero-heating current, while the upper curves have been obtained for several values of the heating current higher than the threshold one. In Figure 4b, the swing amplitudes of the V-Φ characteristics as a function of the heating current are reported. From both Figure 4a,b it is evident that, as the heating current increases, the amplitude of the V-Φ characteristics decreases until it becomes zero for a heating current equal to 8 mA. The asymmetry observed in the curves (Figure 4a) is due to the effect of the APF circuit, which determines an increase in the voltage responsivity, that is, of the derivative value calculated at the point of maximum slope of the curve (V_Φ_ = ∂V/∂Φ_e_). In such a way, if the working point is chosen in the steepest region of the characteristic, a reduction in the noise contribution of the readout amplifier is obtained.

From a quantitative point of view, we can write the voltage and intrinsic responsivity [32]:

(2) $$\Delta V=\frac{\alpha }{\pi }\frac{4-\alpha }{1+\beta_{\mathrm{eff}}}I_{c}R;\;V_{\Phi }^{\mathrm{int}}=\frac{4a}{1+\beta_{\mathrm{eff}}}\frac{I_{c}R}{\Phi_{0}};\;\left(\alpha =1-2\frac{\sqrt{\pi k_{B}TL}}{\Phi_{0}}\right)$$

and APF responsivity and gain as [34]:

(3) $$V_{\Phi }^{\mathrm{APF}}=\frac{1}{1-G_{APF}}V_{\Phi }^{\mathrm{int}};\;G_{\mathrm{APF}}=V_{\Phi }^{\mathrm{int}}\frac{M_{\mathrm{APF}}}{R_{\mathrm{APF}}}$$

where *G*_APF_ is the APF gain, M_APF_ is the mutual inductance between the SQUID loop and APF coil, R_APF_ is the resistance of APF circuit, and k_B_ is the Boltzmann constant. If the critical current is adjusted by local heating of the sample, ∆V decreases as well as the intrinsic responsivity (*V*_Φ_^int^) since they depend (directly) on the *I*_c_ value. As a consequence, also the effect of the APF circuit on the responsivity becomes less effective because the *V*_Φ_^APF^ approaches the intrinsic one. This is reflected in lesser asymmetry of the V-Φ characteristics, as can be seen from Figure 4a. Note that even if the APF circuit is designed to have a gain suitably smaller than the unity to avoid unstable working conditions, the G_APF_ can assume value very close to unity leading the sensor to exhibit a high magnetic noise value.

This can occur when the critical current has a value greater than the expected one, leading to an increase in the intrinsic responsivity as well as the APF gain which assume a too high value. In this circumstance, the local heating procedure can be also exploited, lowering the critical current by a suitable amount sending an appropriate heating current in order to obtain a more suitable voltage behavior as a function of magnetic flux.

**Figure 5:**
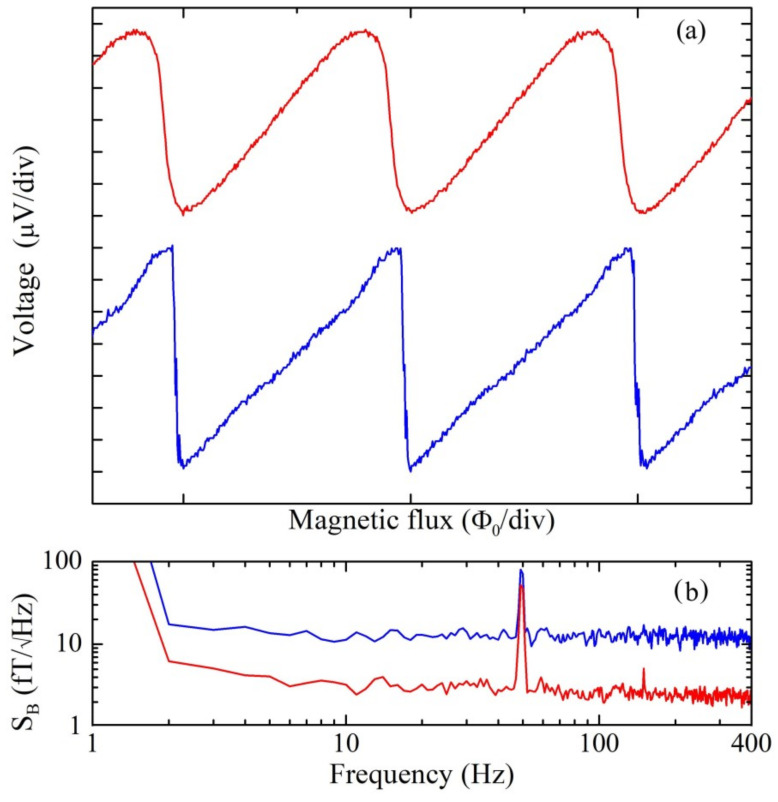
(**a**) Voltage–magnetic flux characteristics of a SQUID magnetometer having a non-optimal critical current value (blue curve) and after sending a current of 6.5 mA into the integrated heater (red curve). (**b**) Spectral density of magnetic field noise of the same sensor before (blue trace) and during the local heating (red trace). These measurements were performed at liquid helium temperature.

Figure 5a shows the V-Φ characteristic (blue/lower curve) of a magnetometer with an APF gain close to one in which a clear noise was observed on the curve and also confirmed by the spectral density of magnetic flux noise reported in Figure 5b (blue /lower trace). In particular, the V-Φ characteristic becomes like a sawtooth causing instability and high noise level especially when the sensor works in FLL mode. In order to optimize this magnetometer, a current of 6.5 mA was sent in the integrated heaters decreasing the critical current of about 20% (passing from 32 mA to 26 mA). As a consequence, the APF gain went below the instability conditions, and the V-Φ characteristic (red/upper curve, Figure 5a) became less noisy leading to a lower spectral density of magnetic field noise that decreases down to 3 fT/√Hz with respect to uncorrected value of 20 fT/Hz^1/2^ (red/lower trace and blue/upper trace in Figure 5b, respectively). In case the critical current tuning is not necessary, i.e., when shunt resistance and critical current values are optimized, the magnetic field spectral density, which is the most important characteristic for a SQUID magnetometer, is typically between 2 and 3 fT/Hz^1/2^. As can be seen from Figure 5, if the critical current is not optimized, the magnetic field noise can be even 7 times greater than the noise of the tuned one whose value is almost equal to that of a magnetometer which does not require tuning.

It is worth noting that the same effect can be obtained by increasing the bias current, thus decreasing the amplitude of the V-Φ and therefore also the responsivity, V_Φ_. As a consequence, the APF gain decreases below 1 and the magnetic flux noise decreases. However, this procedure is not very effective when the I_bias_ to I_C_ ratio is high (above 15–20%). In this case, in addition to moving from the optimal I_bias_ value (I_bias_ = 2.1 I_C_), which minimizes the noise, the magnetic noise obtained with the aforementioned procedure does not fall below 6–7 fT/Hz^1/2^ and above all, in most cases, an unwanted low-frequency noise appears which renders the devices unusable for magnetoencephalography measurements.

The smoother V-Φ characteristic (red/upper curve of Figure 5a) guarantees the necessary stability of operation especially when the sensor works in FLL mode. In fact, if the working point is locked in a very sloping region of the V-Φ characteristic, the slew rate decreases and an impulse due to external noise or a fast signal can lead to get out from the FLL mode. Note that, compared to standard thermal annealing techniques which require high temperature heating out of the measurement stage [36], the effect is fully reversible and can be performed as needed under operating conditions.

## 4. Conclusions

In conclusion, the experimental results concerning a tunable SQUID magnetometer are reported. The possibility to modify, in a stable and reliable way, the critical current and the performance by means of the integration of an appropriate heating resistor on the same chip of a highly sensitive magnetometer has been shown. Such operations are fully reversible and represent an effective tool to tune all parameters which depend on the I_c_ value. In addition, the heating can be forced to above the critical temperature of the superconductor, inducing a temporary transition to the normal state to eliminate the possible magnetic flux entrapped without lifting the device out of the helium bath. This opportunity is particularly useful in multichannel systems like those for biomagnetism in which the single sensor heating cannot be performed without warming up the whole system.

A practical example is reported where the heating operation is performed on a SQUID magnetometer having a non-ideal feature together with a high magnetic field noise improving the performance until it exhibits enhanced characteristics and significantly lower noise.

## Data Availability

Not applicable.
